# Enhancing the Magnetic Behaviors of Dy_2_ Complexes by Modulating the Crystal Field Environment with Different *μ*-O Bridging Ligands

**DOI:** 10.3390/molecules30061260

**Published:** 2025-03-11

**Authors:** Xirong Wang, Min Zhou, Wen Wang, Fangting Zhu, Shijia Qin, Xiulan Li, Feifei Bai, Qinglun Wang, Licun Li, Yue Ma, Bin Zhao

**Affiliations:** Frontiers Science Center for New Organic Matter, Key Laboratory of Advanced Energy Materials Chemistry (MOE), College of Chemistry, Nankai University, Tianjin 300071, China; 2120231054@mail.nankai.edu.cn (X.W.); 2120220968@mail.nankai.edu.cn (M.Z.); 1120230444@mail.nankai.edu.cn (W.W.); 2120220952@mail.nankai.edu.cn (F.Z.); 2120231050@mail.nankai.edu.cn (S.Q.); 2120231061@mail.nankai.edu.cn (X.L.); bfnankai@163.com (F.B.); wangql@nankai.edu.cn (Q.W.); llicun@nankai.edu.cn (L.L.)

**Keywords:** dinuclear lanthanide complexes, magnetization relaxation, single molecule magnets

## Abstract

Four similar dinuclear lanthanide complexes have been synthesized by linking two [Ln(hfac)_2–3_] units (hfac stands for hexafluoroacetylacetone) with different *μ*-O bridging ligands. The 2,2′-bipyridine-N-oxide ligand (bmpo) constructed two centrosymmetric complexes [Ln_2_(hfac)_6_(bmpo)_2_] (Ln = Dy(**1**), Tb(**2**)), with nine-coordinated Ln^III^ ions showing *C*_s_ low symmetry, while the ligand di(2-pyridyl)methanediol (py_2_C(OH)_2_) formed another two compounds [Ln_2_(hfac)_4_(py_2_C(OH)O)_2_] (Ln = Dy(**3**), Tb(**4**)), with two kinds of eight-coordinated Ln^III^ ions exhibiting improved symmetries of *D*_4d_ and *D*_2d._ Magnetic analysis reveals that Dy_2_ complex **1** shows intramolecular antiferromagnetic coupling (*J* = −1.07 cm^−1^) and no relaxation process above 2.0 K even in a 1000 Oe dc field, owing to the low symmetry of Dy^III^ ions, while the similar Dy_2_ complex **3** with improved Dy^III^ symmetry shows ferromagnetic coupling (*J* = 1.17 cm^−1^), which induces a 1000 Oe dc field-induced two-step magnetization relaxation processes with effective energy barrier *U*_eff_ = 47.4 K and 25.2 K for the slow relaxation and fast relaxation processes, respectively. This study proves again that the improved symmetry combined with intramolecular ferromagnetic interactions, both mediated by bridging ligands, can enhance the Dy^III^ anisotropy, further quench the quantum tunneling of the magnetization, and finally, enhance the magnetic behavior of Ln^III^-based systems.

## 1. Introduction

With the rapid development and popularity of artificial intelligence, high density information storage has once again become a hot research topic. And among all storage materials, single-molecule magnets (SMMs) with magnetic bistability and slow magnetization relaxation phenomena at the molecular scale have attracted much attention [1] based on their promising application prospects for high-density information storage [2], molecular spintronics [3,4], and quantum computing [5,6,7]. Ln^III^ ions (notably Dy^III^ ion) with significant magnetic anisotropy are good candidates for constructing high-performance SMMs with higher effective energy barriers (*U*_eff_) and blocking temperatures (*T*_B_) [8,9,10]. However, Ln^III^ ions also have the inherent defect of rapid quantum tunneling of magnetization (QTM), which always limits the relaxation barrier and working temperature. In an attempt to effectively suppress QTM and enhance the energy barrier, one strategy is to adjust the coordination geometry around Ln^III^ ions by especially improving the crystal field symmetries to be as high as those of *C*_∞v_, *S*_8_, *D*_4d_, *D*_5h_, and *D*_6d_ [11,12]. Alternatively, introducing other spin carriers (such as 4f, 2p, and 3d) to induce the intramolecular coupling interactions can also affect the energy levels and suppress QTM [13,14,15,16]. Up to now, a large number of mononuclear [17,18,19,20,21,22] and multinuclear Ln-SMMs [23,24,25,26] have been reported. The representative report of dysprosium metallocene SMMs [Dy(Cp^ttt^)_2_][B(C_6_F_5_)_4_] shows the blocking temperature up to 60 K [20]. Subsequently, a more linear heteroligand analog [(Cp^iPr5^)Dy(Cp*)][B(C_6_F_5_)_4_] increased the record *T*_B_ to 80 K [17]. These breakthroughs have inspired more researchers to further study Ln-SMMs in order to further increase the *T*_B_ and *U*_eff_.

Except for single-ion magnets, dinuclear complexes provide the simplest model to investigate the influence of magnetic interactions on the slow relaxation behavior of Dy^III^-based systems [27,28]. The bridging ligand might mediate both crystal field symmetries and superexchange pathways, which may significantly impact the magnetic behavior [29]. Furthermore, intramolecular magnetic coupling—both ferromagnetic and antiferromagnetic—can suppress QTM to some extent [30,31]. Notably, variations in the local environment at the center of lanthanide systems, in principle, give rise to distinct crystal fields, and even minute alterations in the coordination environment can exert significant influences on the overall magnetic characteristics of SMMs [32,33]. Consequently, the primary strategy for modulating the behavior of SMMs involves the design and modification of ligands, encompassing adjustments to isomers, substitution of ligand substituents, and alteration of terminal ligands, among other approaches [34].

**Scheme 1:**
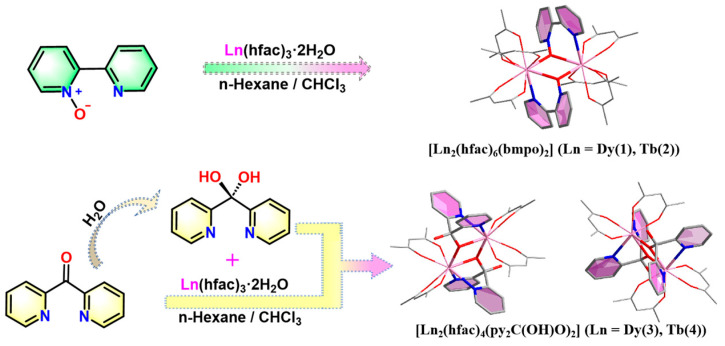
Synthesis of complex **1**–**4** and schematic representation of the different *μ*-O bridging ligands.

In this paper, two different *μ*-O bridging ligands, 2,2′-bipyridine-N-oxide and di(2-pyridyl)methanediol, were utilized to link two [Ln(hfac)_2–3_] units to obtain four similar dinuclear complexes of [Ln_2_(hfac)_6_(bmpo)_2_] (Ln = Dy(**1**), Tb(**2**)) and [Ln_2_(hfac)_4_(py_2_C(OH)O)_2_] (Ln = Dy(**3**), Tb(**4**)), which are illustrated in Scheme 1. Different bridging ligands not only adjusted the crystal field environment and the symmetry of Ln^III^ ions, but also provided different magnetic interactions between Ln^III^ ions. With the increasing coordination symmetry of the Dy^III^ ion and the interaction changing from antiferromagnetic to ferromagnetic coupling, the slow relaxation behavior of complex **3** was improved compared to that of similar complex **1**.

## 2. Results and Discussion

### 2.1. Crystal Structure Description

**Figure 1:**
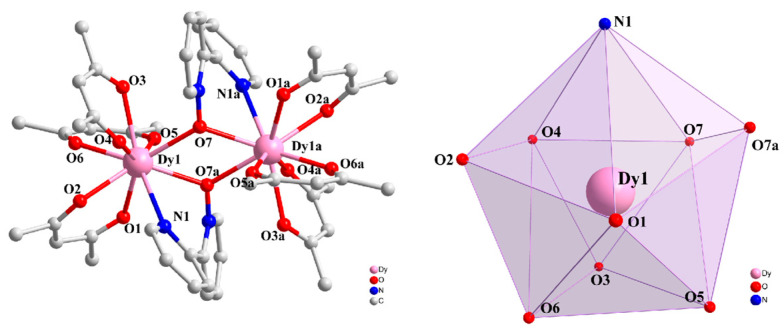
The crystal structure and coordination polyhedron configuration of complex **1** (H and F atoms have been omitted for clarity).

Complexes [Ln_2_(hfac)_6_(bmpo)_2_] (Ln = Dy(**1**), Tb(**2**)) are isomorphic centrosymmetric dinuclear compounds, as revealed by single-crystal X-ray crystallographic analysis. Therefore, only Dy^III^ complex **1** crystallizing in monoclinic crystal system and *C*2/c space group is described in detail. The Dy2 dimer consists of two [Dy(hfac)_3_] units bridged by two oxygen atoms (O7 and O7A) from bridging ligands of 2,2′-bipyridine-N-oxide as in Figure 1. In particular, each Dy^III^ ion is nine-coordinated in a DyNO_8_ coordination environment, formed by a nitrogen atom (N1) and two oxygen atoms (O7 and O7A) from the bmpo ligands, with the remaining six oxygen atoms all originating from three hfac^−^ ligands. The coordination symmetry of Dy^III^ ions is subsequently analyzed using the Shape 2.1 program [35]. The results in Table S1 reveal that the central Dy^III^ ion is located in muffin (*C*_S_) geometry with a CShM value of 0.683. The Dy-O bond distances range from 2.332(4)–2.487(5) Å and the Dy-N bond length is 2.639(6) Å. The O-Dy-O angles fall within the range of 58.30(16)–144.73(17)°, and the Dy1-O7-Dy1a angle is 121.70(16)°. The O-Dy-N angles are within 64.54(16)–144.93(18)°. Figure S1 shows the stacking diagram of complex **1**, which reveals no significant π∙∙∙π stacking or hydrogen bonding interactions between the molecules. The nearest intermolecular Dy∙∙∙Dy distance is 9.9676(2) Å and the intramolecular Dy∙∙∙Dy distance is 4.2760(6) Å. Complex **2** is isomorphic to complex **1**, with only slight deviations in bond lengths and angles compared to complex **1**, due to the different center metal ions.

**Figure 2:**
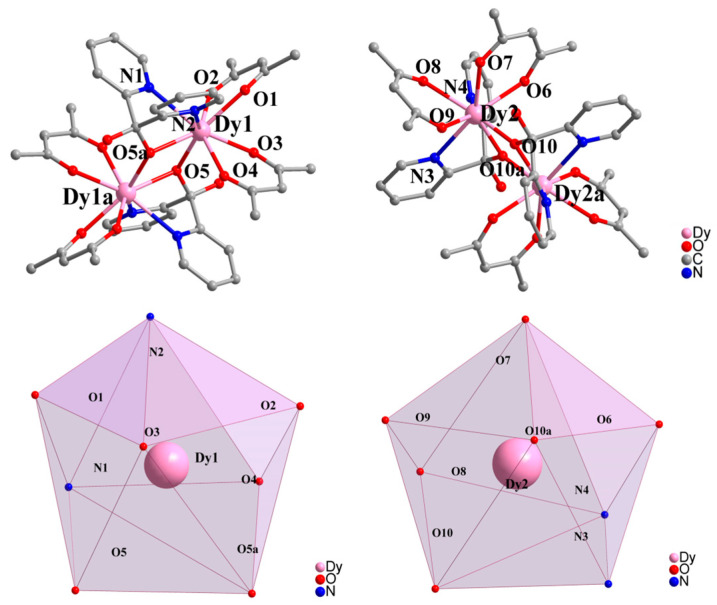
The crystal structure and coordination polyhedron configuration of complex **3** (H and F atoms have been omitted for clarity).

Complexes **3**–**4** are also isostructural, crystallizing in a triclinic crystal system, space group *P*ī, and containing two asymmetric [Ln_2_(hfac)_4_(py_2_C(OH)O)_2_] molecules in one unit cell. Each molecule is a centrosymmetric dinuclear structure consisting of two [Dy(hfac)_2_] moieties connected by two *μ*-O bridging ligands (py_2_C(OH)O)^−^ (Figure 2). The Dy^III^ ions are both eight-coordinated in a DyN_2_O_6_ environment, with two nitrogen atoms of pyridine rings from one (py_2_C(OH)O)^−^ ligand occupying two coordination sites. Additionally, two *μ*-O (O5, O5a) are from two different (py_2_C(OH)O)^−^ ligands and the other four oxygen atoms are from two hfac^−^ ligands. The coordination geometries of center Dy^III^ ions are analyzed using Shape 2.1 software (Table S2). The results indicate that Dy1 is in square antiprismatic configuration with *D*_4d_ symmetry, and Dy2 is in triangular dodecahedron configuration with *D*_2d_ symmetry. The Dy-O bond distances fall between 2.266(5) and 2.424(5) Å. The Dy-N bond distances are between 2.519(6) and 2.552(6) Å. The O-Dy-O angles are within 69.90(17)–151.74(19)°. The Dy-O-Dy angles are 107.45(19)° for Dy1-O5-Dy1 and 109.1(2)° for Dy2-O10-Dy2. The O-Dy-N angles are around 64.42(18)–151.64(18)°. No π∙∙∙π interactions are found in the stacking diagram (Figure S2) but hydrogen bonds exist in the molecules. The shortest intermolecular Dy∙∙∙Dy distance is 9.1431(7) Å. The intramolecular Dy∙∙∙Dy distances is 3.7243(6) Å for Dy1 and 3.7265(8) Å for Dy2. The structure of complex **4** closely resembles that of complex **3**, except for minor differences in bond lengths and angles.

### 2.2. Static Magnetic Characterizations

**Figure 3:**
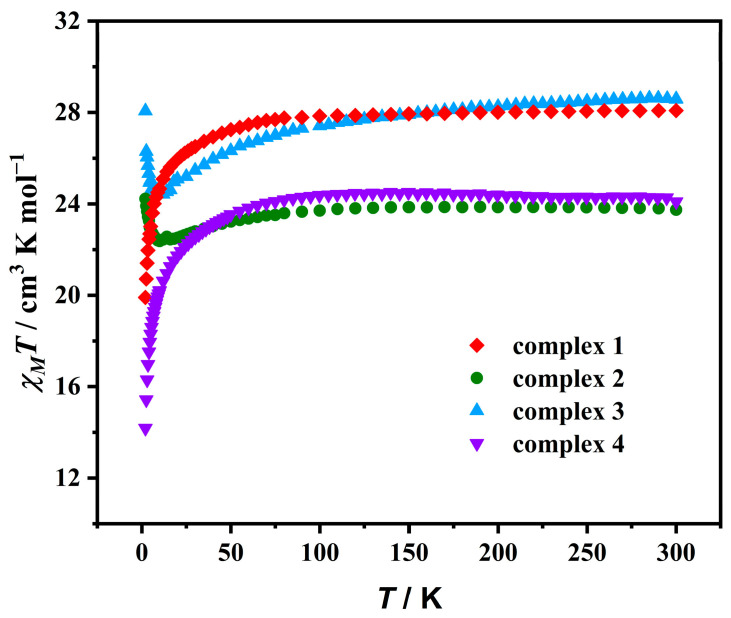
*χ*_M_*T* versus *T* for complexes **1**–**4** at a dc field of 1000 Oe.

We measured the temperature-dependent magnetic susceptibilities of complexes **1**–**4** under a 1000 Oe field between 2 K and 300 K. The *χ*_M_*T* values for Dy2 complexes **1** and **3** are 28.08 and 28.59 cm^3^·K·mol^−1^ (Figure 3), respectively, closely matching 28.34 cm^3^·K·mol^−1^ for two uncoupled Dy^III^ ions (^6^*H*_15/2_, *g* = 4/3). For complex **1**, upon cooling to 30 K, the *χ*_M_*T* value decreases gradually. Further cooling causes it to decrease rapidly, reaching a minimum of 19.90 cm^3^·K·mol^−1^. The reduction of *χ*_M_*T* might be explained by the depopulation of Dy^III^ ions Stark sublevels and/or the intra/intermolecular magnetic interactions between Dy^III^ ions. While for **3**, a decrease is observed of the *χ*_M_*T* from 300 to 12 K, with it reaching 24.42 cm^3^·K·mol^−1^ at 12 K, which is also due to the depopulation of Dy^III^ ions’ Stark sublevels and magnetic coupling. However, cooling down from 12 K to 2 K makes the *χ*_M_*T* increase abruptly to 28.06 cm^3^·K·mol^−1^ at 2 K, which suggests an intramolecular ferromagnetic coupling interaction between two Dy^III^ ions.

The *χ*_M_*T* values of Tb^III^ complexes **2** and **4** at 300 K are 23.75 cm^3^·K·mol^−1^ and 24.09 cm^3^·K·mol^−1^, respectively, close to 23.64 cm^3^·K·mol^−1^ for two uncoupled Tb^III^ ions (^7^*F*_6_, *g* = 3/2), as in Figure 3. The *χ*_M_*T* value for complex **2** has almost no change within the temperature range from 300 to 100 K, and then shows a gradual decrease between 100 and 10 K, arriving at the minimum value of 22.37 cm^3^·K·mol^−1^ at 10 K. Further lowering of the temperature makes the *χ*_M_*T* value begin to increase and eventually reach 24.22 cm^3^·K·mol^−1^ at 2 K, which indicates an intramolecular ferromagnetic interaction between Tb^III^ ions [36]. For complex **4**, the *χ*_M_*T* value remains relatively stable within 300–50 K, then decreases quickly below 50 K and ultimately reaches 14.17 cm^3^·K·mol^−1^ at 2 K.

**Figure 4:**
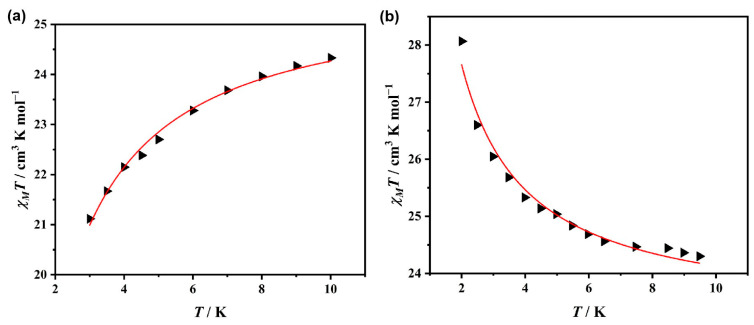
The best-fit curves of the temperature dependence of *χ*_M_*T* for complex **1** (**a**) and **3** (**b**) using the dimeric Ising model.

To study whether the different *μ*-O bridging ligands induce various Dy-Dy interactions, the experiment *χ*_M_*T* vs. *T* data for complexes **1** and **3** at a low temperature range of 2–10 K have been fitted based on the Dy2 dimer Ising model, considering only an effective spin *S* = 1/2 and the anisotropic *g* tensor (Figure 4). At zero magnetic field, when only the *z* components are considered, the theoretical temperature dependence of *χ*_M_*T* is described by Equation (1). The optimal fitting results give *g* = 19.26, *J* = −1.17 cm^−1^ for complex **1** and *g* = 19.28, *J* = 1.07 cm^−1^ for complex **3**. The *g* values are in good agreement with those for a pure *m_J_* = ±15/2 ground state of Dy^III^ ion. The negative *J* value in complex **1** and positive *J* value in complex **3** demonstrate the antiferromagnetic and ferromagnetic Dy-Dy interactions within the molecule mediated by different *μ*-O bridging ligands.

(1) $$\chi_{M}T=(Ng^{2}\beta^{2}/3k)/[1+\exp(-J/2kT)]$$

**Figure 5:**
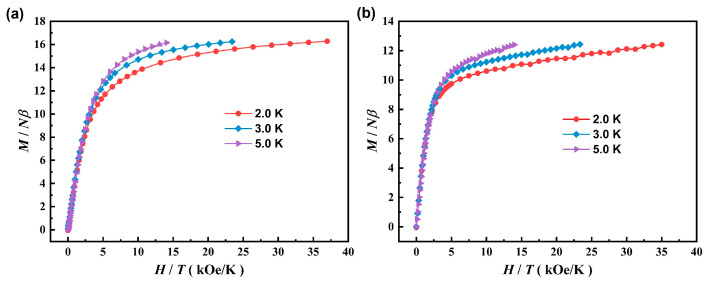
The *M* vs. *H*/*T* plots of complexes **1** (**a**) and **3** (**b**) at different temperatures.

The *M* versus *H*/*T* data were measured from 0 to 70 kOe at various temperatures for Dy^III^ complexes **1** and **3**, as in Figure 5. The *M* values initially increase sharply with increase in magnetic field, then increase slowly, and reach maximum values of 16.28 *μ*_B_ and 12.41 *μ*_B_ at 2 K for complexes **1** and **3,** respectively. Both values are obviously below the anticipated saturation value of 20 *μ*_B_ for two independent Dy^III^ ions (*g*_J_ × *J* = 4/3 × 15/2 = 10 *μ*_B_ for single Dy^III^ ion) for two independent Dy^III^ ions, and the curves of *M* versus *H*/*T* across various temperatures are non-superposable, suggesting the existence of magnetic anisotropy and/or low-lying excited states in the system.

### 2.3. Dynamic Magnetic Measurements

**Figure 6:**
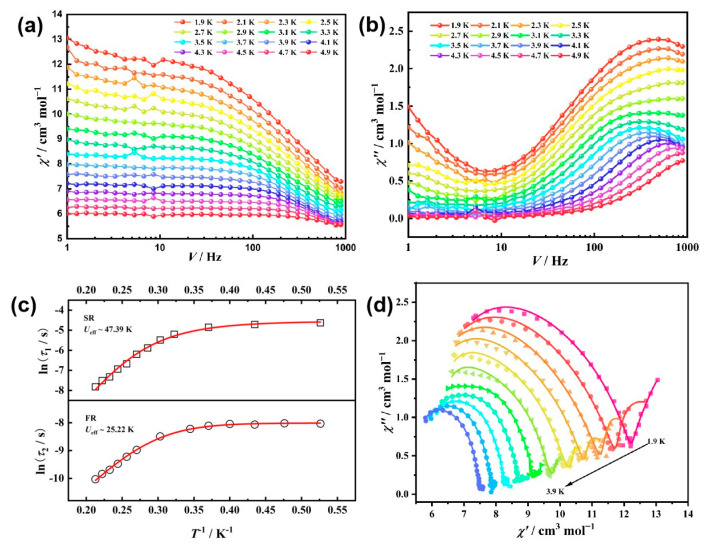
Plots of the frequency-dependent of (**a**) in-phase and (**b**) out-of-phase ac susceptibility for complex **3** under a static magnetic field of 1000 Oe and an oscillating field of 3 Oe, (**c**) ln(*τ*) vs. 1/*T*, and (**d**) Cole–Cole fitting from 1.9 K to 4.9 K for complex **3**.

In order to analyze the dynamics magnetization behavior of Dy^III^ complexes **1** and **3**, their alternating-current (ac) magnetic susceptibilities measurements were performed. For complex **1**, no temperature-dependent out-of-phase (*χ*″) signals are observed at zero direct current (dc) field or even at 1 kOe external field (Figure S3). This may be attributed to the serious QTM, resulting in complex **1** not exhibiting SMM behavior even above 2 K. For complex **3**, as depicted in Figure S4, in a zero dc field, only weak *χ*″ signals are observed, while without appearance of peaks, which also may be caused by QTM. To quench the potential QTM effect, a 1 kOe dc field is used, and both *χ*′ and *χ*″ display significant frequency- and temperature-dependent signals, as shown in Figure S5. In particular, two peaks are detected in *χ”* signals, indicating the dual steps relaxation behavior, which could also be confirmed by the asymmetric semicircle Cole–Cole curves at 1.9–4.9 K (Figure 6d). Therefore, as shown in Figure 6c, two sets of relaxation time (*τ*) and parameters (*α*) are successfully calculated by fitting the frequency-dependent plot using the modified Debye functions model, as in Equation (2). For the slow relaxation (SR) process, the good ln *τ* vs. 1/*T* results are achieved by considering the QTM (*τ*_QTM_ = 0.01 s), Orbach (*U*_eff_/*k*_B_ = 47.39 K and *τ*_0_ = 2.83 × 10^−8^ s), and Raman (*C* = 0.025 s^−1^ K^−n^ and n = 5.85) processes, as given by Equation (3). The range of *α* parameter values is 0.14–0.28. For the fast relaxation (FR) process, the best fitting data is obtained by considering the QTM (*τ*_QTM_ = 3.32 × 10^−4^ s) and Orbach (*U*_eff_/*k*_B_ = 25.22 K and *τ*_0_ = 2.26 × 10^−7^ s) processes. The range of *α* parameter values is 0.25–0.62. The two-step relaxation phenomenon may be due to the different crystal field symmetries of Dy1 and Dy2 in complex **3**. The *U*_eff_/*k*_B_ of complex **3** is higher than some other Dy2 complexes reported in the literature (Table S7).

(2) $$\chi_{ac}\left(\omega \right)=\frac{\chi_{2}-\chi_{1}}{1+{\left(i\omega \tau_{2}\right)}^{\left(1-\alpha_{2}\right)}}+\frac{\chi_{1}-\chi_{0}}{1+{\left(i\omega \tau_{1}\right)}^{\left(1-\alpha_{1}\right)}}+\chi_{0}$$

(3) $$\tau_{1}={\tau_{QTM}}^{-1}+CT^{n}+\tau_{0}\exp\left(-U_{eff}/k_{B}T\right)$$

In parallel, the dynamic magnetic behavior of complexes **2** and **4** under a 0 dc field and at a frequency of 800 Hz (Figure S6) were also investigated. Neither of the two complexes exhibited any *χ*″ signals, even at 2 K. Hence, we conclude that the complexes do not exhibit SMM behavior under the above experimental conditions. This observation may be that the Tb^III^ ion is not a Kramer ion, possessing a relatively small energy barrier that is not sufficient to prevent its spin reversal.

### 2.4. Structure–Property Relationship

**Figure 7:**
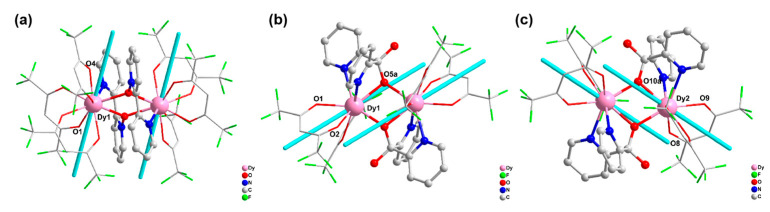
Calculated magnetic anisotropy axes of Dy1 in complex **1** (**a**), Dy1 (**b**), and Dy2 (**c**) in complex **3** using the MAGELLAN program.

Comparing the SMM behaviors of complex **1** and **3**, it is evident that complex **3** shows significant SMM behavior, whereas complex **1** does not display magnetic relaxation behavior under similar experimental conditions. The *μ*-O bridging ligand di(2-pyridyl)methanediol in complex **3** improved the crystal field symmetry of Dy^III^ ions to be *D*_4d_ and *D*_2d_ compared with that of *C*_s_ in complex **1**, which is the main factor for the better SMMs behavior of **3**. Additionally, the magnetic anisotropy axes of the central Dy^III^ ions in complexes **1** and **3** were also calculated (Figure 7) using the Magellan program and were [37] based on the electrostatic model. The magnetic axes of the two Dy^III^ ions must be parallel or antiparallel within the Dy2 dimers of both **1** and **3** based on their centrosymmetric structures. In complex **1**, the magnetic axes are oriented closer to the negatively charged O atoms (O4, O1) from two hfac^−^ ligands (Figure 7a), and the shortest Dy-O bond (Dy1-O4) deviates from the anisotropy axis with an angle of 20.455(88)°, while, in complex **3**, the magnetic axis of Dy1 (Figure 7b) is oriented closer to the negatively charged O atoms (O5a, O1) from deprotonated di(2-pyridyl)methanediol and the hfac^−^ ligand. The magnetic axis is nearly parallel to the shortest Dy1-O5a bond, with a small angle of 11.052(113)°. The smaller angle of the shortest Dy-O bond in complex **3** might induce enhanced axial electron density and reduced equatorial field strength, which will further suppress QTM and enhance SMM properties in complex **3** [38]. The magnetic axis of Dy2 in complex **3** (Figure 7c) deviates slightly from Dy2-O10a, with an angle of 21.579(137)°. However, the overall coordination environment still favors better SMM behavior in complex **3**.

In addition, the different Dy-Dy interactions mediated by the different *μ*-O bridging ligands might also affect the magnetic behaviors. The magnetic coupling interactions between the parallel moments within the dimer are simplified by Equation (4) [39,40], where *θ* represents the angle between the magnetic axes and the line connecting the two spin centers. When *θ* is below the threshold of 54.75°, the coupling is ferromagnetic; otherwise, it is antiferromagnetic. The *θ* values of 78.113° for complex **1** and 31.534(9)° and 32.180(8)° for complex **3** indicate antiferromagnetic Dy-Dy interactions in complex **1** and ferromagnetic coupling in complex **3**, which are consistent with the *J* values derived from fitting the experimental data with the Ising model.

(4) $$E_{dip}=-\frac{\mu_{0}}{4\pi }\frac{\mu^{2}}{r^{3}}[3\cos^{2}\theta -1]$$

In conclusion, the significant difference in SMM behavior between complex **1** and complex **3** could be attributed to the modulation of different *μ*-O bridging ligands. Complex **3** exhibits improved coordination symmetry (*D*_4d_ and *D*_2d_), stronger uniaxial anisotropy and ferromagnetic Dy-Dy interactions, which together suppress QTM effectively and enhance the *U*_eff_ to some extent.

## 3. Experimental Section

All reagents are commercially sourced and utilized directly without additional purification.

### 3.1. Materials and Physical Techniques

The 2,2′-bipyridine-N-oxide (bmpo) was synthesized following the literature approach [41]. [Ln(hfac)_3_]·2H_2_O (Ln = Tb^III^, Dy^III^) have been synthesized with the related methods [42]. Di(2-pyridyl)ketone was bought from the Energy Chemical company (Shanghai, China). Elemental analyses (C, H, and N) were conducted using a Vario EL cube (Langenselbold, German). The analysis of IR spectra was performed on a Nicolet iS10 ATR-FTIR instrument (Thermo, Waltham, MA, USA), spanning from 4000 to 400 cm^−1^. Magnetic susceptibility measurements were tested on a Quantum Design MPMS-7 SQUID magnetometer (Santa Clara, CA, USA). The diamagnetic corrections of all constituent atoms were performed on Pascal’s constants [43,44,45].

### 3.2. X-Ray Crystallography

Complexes **1**–**4** were measured on an Oxford SuperNova TM diffractometer (San Diego, CA, USA) using Mo-K*α* monochromatic radiation (*λ* = 0.71073 Å). The structural data were solved by the direct method, and analytical refinement was performed using the SHELXS-2014 [46], SHELXL-2014 [47] and Olex2-1.2 software package. Anisotropic refinement was performed on *F*^2^ by the full-matrix least-squares method for all non-hydrogen atoms. Owing to the high level of disorder in the solvent molecules, the scattering contributions from the disordered C_7_H_16_ component were masked via the SQUEEZE routine in Olex2 [48,49]. Table 1 summarizes the detailed crystallographic parameters of complexes **1**–**4**. Tables S3–S6 list some of the important bond lengths and angles for complexes **1**–**4**. CCDC number: 2405439–2405440 for **1**–**2**, 1999479–1999480 for **3**–**4**.

### 3.3. Synthesis of [Ln_2_(hfac)_6_(bmpo)_2_] (Ln = Dy(1), Tb(2))

[Dy(hfac)_3_]·2H_2_O (0.05 mmol, 0.0408 g) was added to 20 mL of n-hexane and refluxed at 100 °C for 2 h. Following this, the temperature was lowered to approximately 70 °C. The aforementioned solution was mixed with a solution of 2,2′-bipyridine-N-oxide (0.05 mmol) dissolved in CHCl_3_, and the mixture was constantly agitated for about 0.5 h at this temperature. Finally, the solution was permitted to reach ambient temperature naturally and then filtered. The resulting filtrate was left to stand for about four days, yielding colorless bulk crystals that are suitable for X-ray analysis. Complex **2** can be prepared by replacing [Dy(hfac)_3_]·2H_2_O with [Tb(hfac)_3_]·2H_2_O using a similar method. Anal. Calcd. for C_50_H_22_Tb_2_F_36_N_4_O_14_ (1): C, 31.53; H, 1.16; N, 2.94%. Found: C, 31.50; H, 1.43; N, 2.87%. IR (KBr cm^−1^): 1652(s), 1504(m), 1251(s), 1191(s), 1135(vs), 1005(w), 950(w), 843(w), 793(s), 773(s), 728(m), 660(s). Anal. Calcd. for C_50_H_22_Dy_2_F_36_N_4_O_14_ (2): C, 31.41; H, 1.15; N, 2.93%. Found: C, 31.49; H, 1.56; N, 2.89%. IR (KBr cm^−1^): 1651(s), 1505(m), 1252(s), 1194(s), 1135(vs), 1005(w), 951(w), 843(m), 793(s), 773(s), 728(m), 659(s).

### 3.4. Synthesis of [Ln_2_(hfac)_4_(py_2_C(OH)O)_2_] (Ln = Dy(3), Tb(4))

Complexes **3**–**4** were synthesized in the same way as complexes **1**–**2** except that di(2-pyridyl) ketone was used instead of 2,2′-bipyridine-N-oxide. Since reversible addition reactions between water and alcohols with carbonyl compounds result in the formation of hydrates (gem-diol compounds) [50,51,52], it is possible that H_2_O in the solvent may convert di(2-pyridyl)ketone into di(2-pyridyl)methanediol during the reaction process, which can be further coordinated to the metal. Further syntheses of mono- and polynuclear transition metal complexes based on hydrolysis and alcoholysis products were reported [53,54,55]. Anal. Calcd. for C_42_H_22_Tb_2_F_24_N_4_O_12_ (3): C, 32.58; H, 1.43; N, 3.62%; Found: C, 32.47; H, 1.51; N, 3.55%. IR (KBr cm^−1^): 1652(vs), 1475(vs), 1250(s), 1194(s), 1132(s), 1035(m), 797(s), 754(s). Anal. Calcd. for C_42_H_22_Dy_2_F_24_N_4_O_12_ (4): C, 32.43; H, 1.42; N, 3.60%; Found: C, 32.49; H, 1.38; N, 3.63%. IR (KBr cm^−1^): 1645(vs), 1472(vs), 1251(s), 1192(vs), 1132(s), 1042(m), 793(s), 755(s).

## 4. Conclusions

To explore how different bridging ligands affect crystal field symmetries and superexchange pathways and ultimately influence the magnetic behaviors of Ln-based SMMs, four similar Ln2 dinuclear complexes were synthesized by utilizing different *μ*-O bridging ligands and hfac^−^ ancillary ligands. The bmpo links two [Ln(hfac)_3_] units to construct two Ln^III^ complexes **1**–**2** with *C*_s_ low symmetry, where complex **1** exhibits antiferromagnetic Dy-Dy interactions and shows no SMMs behavior even under a 1000 Oe dc field. When the bridging ligand is changed to di(2-pyridyl) methanediol, it improves the symmetry of the Ln^III^ ions in complexes **3**–**4** to *D*_4d_ and *D*_2d_, with complex **3** exhibiting ferromagnetic Dy-Dy interactions that induce a two-step relaxation process in a 1000 Oe dc field with *U*_eff_ = 47.4 K for the SR relaxation and *U*_eff_ = 25.2 K for the FR relaxation, respectively. This study proves again that suitable bridging ligands can improve symmetry, and when combined with intramolecular ferromagnetic interactions, they can enhance the Dy^III^ anisotropy, further quench the QTM, and finally enhance the magnetic behavior of Dy^III^-based systems.

## Figures and Tables

**Table 1:** Crystallographic data of **1**–**4**.

	1	2	3	4
Empirical formula	C_50_H_22_Dy_2_F_36_N_4_O_14_	C_50_H_22_Tb_2_F_36_N_4_O_14_	C_42_H_22_Dy_2_F_24_N_4_O_12_	C_42_H_22_Tb_2_F_24_N_4_O_12_
Formula weight	1911.71	1904.56	1555.63	1548.47
Temperature/K	300.01(10)	120.00(10)	137.00	113.00
Crystal system	monoclinic	monoclinic	triclinic	triclinic
Space group	*C*2/c	*C*2/c	*P*ī	*P*ī
*a* [Å]	22.3250(4)	22.2755(3)	11.8996(9)	11.966(2)
*b* [Å]	15.5402(3)	15.2108(3)	12.0756(10)	12.126(2)
*c* [Å]	19.1924(3)	18.9123(3)	20.9612(16)	21.023(4)
*a* [deg]	90	90	76.529(7)	76.41(3)
*b* [deg]	99.915(2)	99.5091(16)	78.452(6)	78.68(3)
*g* [deg]	90	90	61.314(8)	61.32(3)
Volume [Å^3^]	6559.1(2)	6319.95(19)	2555.8(4)	2588.7(12)
*Z*	4	4	2	2
*ρ*_calc_ [g/cm^−3^]	1.936	2.002	2.021	1.987
*μ* [mm^−1^]	13.586	12.455	3.052	2.859
*F*(000)	3672	3664	1492	1488
Reflections collected	19,975	24,225	17,394	23,369
Unique/parameters	6536/478	6351/478	8984/758	9102/936
*R*(int)	0.0882	0.0430	0.0497	0.1232
Goodness-of-fit on *F*^2^	1.028	1.073	1.085	0.981
*R*_1_, w*R*_2_ [*I* > 2*σ*(*I*)]	0.0772, 0.2127	0.0477, 0.1211	0.0512, 0.0960	0.0783, 0.1740
*R*_1_, w*R*_2_ (all data)	0.0873, 0.2205	0.0554, 0.1250	0.0736, 0.1151	0.0988, 0.1908

## Data Availability

Data are available on request from the authors.

## Supplementary Materials

The following supporting information can be downloaded at: https://www.mdpi.com/article/10.3390/molecules30061260/s1, Figure S1: Crystal packing diagram of complex **1** (H and F atoms are omitted for clarity); Figure S2: Crystal packing diagram of complex **3** (H and F atoms are omitted for clarity); Figure S3: (a) temperature dependence of out-of-phase (*χ*″) ac magnetic susceptibilities for **1** under zero dc fields and (b) under 1 kOe fields; Figure S4: temperature dependence of in-phase(*χ*′) and out-of-phase (*χ*″) ac magnetic susceptibilities under zero dc fields for **3**; Figure S5: (a,b) temperature dependence and (c,d) frequency dependence of in-phase(*χ*′) and out-of-phase (*χ*″) ac magnetic susceptibilities under 1 kOe fields for **3**; Figure S6: (a) temperature dependence of the *χ*′ and *χ*″ ac magnetic susceptibility in 800 Hz under zero dc fields for **2** and (b) for **4**; Table S1: Dy^III^ geometry analysis of complex **1** by using SHAPE 2.1 software; Table S2: Dy^III^ geometry analysis of complex **3** by using SHAPE 2.1 software; Table S3–S6: Some of the important bond lengths [Å] and angles [°] for complex **1**–**4**; Table S7. Some reported Dy2 complexes exhibiting SMM behavior [33,56,57,58,59,60,61,62,63,64].

## References

[1] Coronado E. (2020). Molecular magnetism: From chemical design to spin control in molecules, materials and devices. Nat. Rev. Mater..

[2] Mannini M., Pineider F., Sainctavit P., Danieli C., Otero E., Sciancalepore C., Talarico A.M., Arrio M.-A., Cornia A., Gatteschi D. (2009). Magnetic memory of a single-molecule quantum magnet wired to a gold surface. Nat. Mater..

[3] Bogani L., Wernsdorfer W. (2008). Molecular spintronics using single-molecule magnets. Nat. Mater..

[4] Troiani F., Affronte M. (2011). Molecular spins for quantum information technologies. Chem. Soc. Rev..

[5] Aromí G., Aguilà D., Gamez P., Luis F., Roubeau O. (2012). Design of magnetic coordination complexes for quantum computing. Chem. Soc. Rev..

[6] Timco G.A., Faust T.B., Tuna F., Winpenny R.E.P. (2011). Linking heterometallic rings for quantum information processing and amusement. Chem. Soc. Rev..

[7] Leuenberger M.N., Loss D. (2001). Quantum computing in molecular magnets. Nature.

[8] Habib F., Murugesu M. (2013). Lessons learned from dinuclear lanthanide nano-magnets. Chem. Soc. Rev..

[9] Ishikawa N., Sugita M., Ishikawa T., Koshihara S.-y., Kaizu Y. (2003). Lanthanide Double-Decker Complexes Functioning as Magnets at the Single-Molecular Level. J. Am. Chem. Soc..

[10] Powell A.K., Layfield R.A., Murugesu M. (2015). Lanthanides and Actinides in Molecular Magnetism.

[11] Liu J.-L., Chen Y.-C., Tong M.-L. (2018). Symmetry strategies for high performance lanthanide-based single-molecule magnets. Chem. Soc. Rev..

[12] Canaj A.B., Dey S., Martí E.R., Wilson C., Rajaraman G., Murrie M. (2019). Insight into D6 Symmetry: Targeting Strong Axiality in Stable Dysprosium(III) Hexagonal Bipyramidal Single-Ion Magnets. Angew. Chem. Int. Ed..

[13] Swain A., Sharma T., Rajaraman G. (2023). Strategies to quench quantum tunneling of magnetization in lanthanide single molecule magnets. Chem. Commun..

[14] Mannini M., Pineider F., Danieli C., Totti F., Sorace L., Sainctavit P., Arrio M.A., Otero E., Joly L., Cezar J.C. (2010). Quantum tunnelling of the magnetization in a monolayer of oriented single-molecule magnets. Nature.

[15] Dey A., Bag P., Kalita P., Chandrasekhar V. (2021). Heterometallic CuII–LnIII complexes: Single molecule magnets and magnetic refrigerants. Coord. Chem. Rev..

[16] Benelli C., Gatteschi D. (2002). Magnetism of Lanthanides in Molecular Materials with Transition-Metal Ions and Organic Radicals. Chem. Rev..

[17] Guo F.-S., Day B.M., Chen Y.-C., Tong M.-L., Mansikkamäki A., Layfield R.A. (2018). Magnetic hysteresis up to 80 kelvin in a dysprosium metallocene single-molecule magnet. Science.

[18] Zhu Z., Zhao C., Feng T., Liu X., Ying X., Li X.-L., Zhang Y.-Q., Tang J. (2021). Air-Stable Chiral Single-Molecule Magnets with Record Anisotropy Barrier Exceeding 1800 K. J. Am. Chem. Soc..

[19] Liu J., Chen Y.-C., Liu J.-L., Vieru V., Ungur L., Jia J.-H., Chibotaru L.F., Lan Y., Wernsdorfer W., Gao S. (2016). A Stable Pentagonal Bipyramidal Dy(III) Single-Ion Magnet with a Record Magnetization Reversal Barrier over 1000 K. J. Am. Chem. Soc..

[20] Goodwin C.A.P., Ortu F., Reta D., Chilton N.F., Mills D.P. (2017). Molecular magnetic hysteresis at 60 kelvin in dysprosocenium. Nature.

[21] Jiang S.-D., Wang B.-W., Su G., Wang Z.-M., Gao S. (2010). A Mononuclear Dysprosium Complex Featuring Single-Molecule-Magnet Behavior. Angew. Chem. Int. Ed..

[22] Zanella S., Aragon-Alberti M., Brite C.D.S., Salles F., Carlos L.D., Long J. (2023). Luminescent Single-Molecule Magnets as Dual Magneto-Optical Molecular Thermometers. Angew. Chem. Int. Ed..

[23] Bajaj N., Mavragani N., Kitos A.A., Chartrand D., Maris T., Mansikkamäki A., Murugesu M. (2024). Hard single-molecule magnet behavior and strong magnetic coupling in pyrazinyl radical-bridged lanthanide metallocenes. Chem.

[24] Deng W., Wu S.-G., Ruan Z.-Y., Gong Y.-P., Du S.-N., Wang H.-L., Chen Y.-C., Zhang W.-X., Liu J.-L., Tong M.-L. (2024). Spin-State Control in Dysprosium(III) Metallacrown Magnets via Thioacetal Modification. Angew. Chem. Int. Ed..

[25] Zhang P., Luo Q.-C., Zhu Z., He W., Song N., Lv J., Wang X., Zhai Q.-G., Zheng Y.-Z., Tang J. (2023). Radical-Bridged Heterometallic Single-Molecule Magnets Incorporating Four Lanthanoceniums. Angew. Chem. Int. Ed..

[26] Fernandez Garcia G., Guettas D., Montigaud V., Larini P., Sessoli R., Totti F., Cador O., Pilet G., Le Guennic B. (2018). A Dy4 Cubane: A New Member in the Single-Molecule Toroics Family. Angew. Chem. Int. Ed..

[27] Guo M., Wang Y., Wu J., Zhao L., Tang J. (2017). Structures and magnetic properties of dysprosium complexes: The effect of crystallization temperature. Dalton Trans..

[28] Tian J., Du J., Li B., Zhang H., Zhang Y., Sun L., Ma P. (2024). Recent advances of dinuclear dysprosium-based single-molecule magnets: From mechanisms to application. J. Mater. Chem. C.

[29] Matheson B.E., Dais T.N., Donaldson M.E., Rowlands G.J., Plieger P.G. (2023). The importance of second sphere interactions on single molecule magnet performance. Inorg. Chem. Front..

[30] Long J., Habib F., Lin P.-H., Korobkov I., Enright G., Ungur L., Wernsdorfer W., Chibotaru L.F., Murugesu M. (2011). Single-Molecule Magnet Behavior for an Antiferromagnetically Superexchange-Coupled Dinuclear Dysprosium(III) Complex. J. Am. Chem. Soc..

[31] Guo Y.-N., Xu G.-F., Wernsdorfer W., Ungur L., Guo Y., Tang J., Zhang H.-J., Chibotaru L.F., Powell A.K. (2011). Strong Axiality and Ising Exchange Interaction Suppress Zero-Field Tunneling of Magnetization of an Asymmetric Dy2 Single-Molecule Magnet. J. Am. Chem. Soc..

[32] Wang M., Guo Y., Han Z., Cheng X., Zhang Y.-Q., Shi W., Cheng P. (2022). Impact of Ligand Substituents on the Magnetization Dynamics of Mononuclear DyIII Single-Molecule Magnets. Inorg. Chem..

[33] Zhang X.-M., Duan Y.-Y., Gao H.-L., Cui J.-Z. (2020). Solvent-induced single-molecule magnet behavior and near-infrared luminescence properties of rare earth complexes. New J. Chem..

[34] Huang Y., Li J.-X., Ge Y., Zhang X.-M., Xu Y., Li Y., Zhang Y.-Q., Yao J.-L. (2020). Designing asymmetric Dy2 single-molecule magnets with two-step relaxation processes by the modification of the coordination environments of Dy(iii) ions. Dalton Trans..

[35] Sheldrick G.M. (2014). SHELXL-2014, Program for Refinement of Crystal Structures.

[36] Sessoli R., Powell A.K. (2009). Strategies towards single molecule magnets based on lanthanide ions. Coord. Chem. Rev..

[37] Chilton N.F., Collison D., McInnes E.J.L., Winpenny R.E.P., Soncini A. (2013). An electrostatic model for the determination of magnetic anisotropy in dysprosium complexes. Nat. Commun..

[38] Rinehart J.D., Long J.R. (2011). Exploiting single-ion anisotropy in the design of f-element single-molecule magnets. Chem. Sci..

[39] Leng J.-D., Liu J.-L., Lin W.-Q., Gómez-Coca S., Aravena D., Ruiz E., Tong M.-L. (2013). Unprecedented ferromagnetic dipolar interaction in a dinuclear holmium(iii) complex: A combined experimental and theoretical study. Chem. Commun..

[40] Guo M., Zhang Y.-Q., Zhu Z., Tang J. (2018). Dysprosium Compounds with Hula-Hoop-like Geometries: The Influence of Magnetic Anisotropy and Magnetic Interactions on Magnetic Relaxation. Inorg. Chem..

[41] Karbakhsh Ravari A., Pineda-Galvan Y., Huynh A., Ezhov R., Pushkar Y. (2020). Facile Light-Induced Transformation of [Ru^II^(bpy)_2_(bpyNO)]^2+^ to [Ru^II^(bpy)_3_]^2+^. Inorg. Chem..

[42] Richardson M.F., Wagner W.F., Sands D.E. (1968). Rare-earth trishexafluoroacetylacetonates and related compounds. J. Inorg. Nucl. Chem..

[43] Zhong L., Chen W.-B., OuYang Z.-J., Yang M., Zhang Y.-Q., Gao S., Schulze M., Wernsdorfer W., Dong W. (2020). Unprecedented one-dimensional chain and two-dimensional network dysprosium(iii) single-molecule toroics with white-light emission. Chem. Commun..

[44] Wang H.-S., Chen Y., Hu Z.-B., Yin C.-L., Zhang Z., Pan Z.-Q. (2019). Modulation of the directions of the anisotropic axes of DyIII ions through utilizing two kinds of organic ligands or replacing DyIII ions by FeIII ions. CrystEngComm.

[45] Sheldrick G.M. (2014). SHELXS-2014, Program for Solution of Crystal Structures.

[46] Llunell M., Casanova D., Cirera J., Alemany P., Alvarez S. (2013). SHAPE 2.1.

[47] Wang H.-S., Zhang K., Song Y., Pan Z.-Q. (2021). Recent advances in 3d-4f magnetic complexes with several types of non-carboxylate organic ligands. Inorganica Chim. Acta.

[48] Dolomanov O.V., Bourhis L.J., Gildea R.J., Howard J.A.K., Puschmann H. (2009). OLEX2: A complete structure solution, refinement and analysis program. J. Appl. Crystallogr..

[49] Rees B., Jenner L., Yusupov M. (2005). Bulk-solvent correction in large macromolecular structures. Acta Crystallogr. Sect. D.

[50] Hine J., Green L.R., Meng P.C., Thiagarajan V. (1976). Structural effects on rates and equilibriums. XX. Rates and equilibriums in hydration and bisulfate addition by 1,3-dimethoxyacetone. J. Org. Chem..

[51] Kokesh F.C. (1976). The determination by proton nuclear magnetic resonance of the enol, hydrate and keto forms of oxaloacetic acid and its anions. J. Org. Chem..

[52] Sayer J.M. (1975). Hydration of p-nitrobenzaldehyde. J. Org. Chem..

[53] Deveson A.C., Heath S.L., Harding C.J., Powell A.K. (1996). Synthesis and structures of copper(II) anion-bridged aggregates and chains: Control over molecular shape. J. Chem. Soc. Dalton Trans..

[54] Papadopoulos A.N., Tangoulis V., Raptopoulou C.P., Terzis A., Kessissoglou D.P. (1996). First Example of a CuII Polymeric Complex Having a Tetranuclear Repeating Unit with a S = 2 Ground State. Crystal Structure of [Cu_4_(dpk·CH_3_O)_2_Cl_6_]n (dpk·CH_3_OH = Unimethylated Diol of Di-2-pyridyl Ketone). Inorg. Chem..

[55] Parker O.J., Aubol S.L., Breneman G.L. (2000). Crystal structure of a copper stabilized hydrate of di-2-pyridyl ketone. Polyhedron.

[56] Moreno Pineda E., Chilton N.F., Marx R., Dörfel M., Sells D.O., Neugebauer P., Jiang S.-D., Collison D., van Slageren J., McInnes E.J.L. (2014). Direct measurement of dysprosium(III)˙˙˙dysprosium(III) interactions in a single-molecule magnet. Nat. Commun..

[57] Gao F., Wang L., Zhu G.-Z., Liu Y.-H., Yang H., Li X., Yang K. (2019). Controllable syntheses and magnetic properties of novel homoleptic triple-decker lanthanide complexes. Dalton Trans..

[58] Zhang W.-Y., Tian Y.-M., Li H.-F., Chen P., Sun W.-B., Zhang Y.-Q., Yan P.-F. (2016). A series of dinuclear Dy(iii) complexes bridged by 2-methyl-8-hydroxylquinoline: Replacement on the periphery coordinated β-diketonate terminal leads to different single-molecule magnetic properties. Dalton Trans..

[59] Wang W.-M., Zhang H.-X., Wang S.-Y., Shen H.-Y., Gao H.-L., Cui J.-Z., Zhao B. (2015). Ligand Field Affected Single-Molecule Magnet Behavior of Lanthanide(III) Dinuclear Complexes with an 8-Hydroxyquinoline Schiff Base Derivative as Bridging Ligand. Inorg. Chem..

[60] Xiao Z.-X., Miao H., Shao D., Wei H.-Y., Zhang Y.-Q., Wang X.-Y. (2018). Single-molecule magnet behaviour in a dysprosium-triradical complex. Chem. Commun..

[61] Shen F.-X., Pramanik K., Brandão P., Zhang Y.-Q., Jana N.C., Wang X.-Y., Panja A. (2020). Macrocycle supported dimetallic lanthanide complexes with slow magnetic relaxation in Dy2 analogues. Dalton Trans..

[62] Shao D., Sahu P.P., Tang W.-J., Zhang Y.-L., Zhou Y., Xu F.-X., Wei X.-Q., Tian Z., Singh S.K., Wang X.-Y. (2022). A single-ion magnet building block strategy toward Dy2 single-molecule magnets with enhanced magnetic performance. Dalton Trans..

[63] Anastasiadis N.C., Kalofolias D.A., Philippidis A., Tzani S., Raptopoulou C.P., Psycharis V., Milios C.J., Escuer A., Perlepes S.P. (2015). A family of dinuclear lanthanide(iii) complexes from the use of a tridentate Schiff base. Dalton Trans..

[64] Zhao C., Zhu Z., Li X.-L., Tang J. (2022). Air-stable chiral mono- and dinuclear dysprosium singlemolecule magnets: Steric hindrance of hexaazamacrocycles. Inorg. Chem. Front..

