# Supplementary material for: Enhancing the Magnetic Behaviors of Dy2 Complexes by Modulating the Crystal Field Environment with Different μ-O Bridging Ligands
Source: Molecules. 2025 Mar 11;30(6):1260. doi: 10.3390/molecules30061260 (PMC11946584; doi:10.3390/molecules30061260)
Supplement: Supplementary file 1 [file molecules-30-01260-s001.zip › molecules-3503478-Sopporting Information.pdf]

Enhancing the Magnetic Behaviors of Dy_2 Complexes by Modulating the Crystal Field Environment with Different μ-O Bridging Ligands

Xirong Wang , Min Zhou , Wen Wang , Fangting Zhu , Shijia Qin , Xiulan Li , Fefei Bai , Qinglun Wang , Licun Li , Yue Ma* , and Bin Zhao*

Frontiers Science Center for New Organic Matter, Key Laboratory of Advanced Energy Materials Chemistry (MOE), College of Chemistry, Nankai University, Tianjin 300071, China

S1. Supporting Figures **1**

Figure S1 Crystal packing diagram of complex **1** (H and F atoms are omitted for clarity). 2

Figure S2 Crystal packing diagram of complex **3** (H and F atoms are omitted for clarity). 2

Figure S3 (a)Temperature dependence of out-of phase (χ'') ac magnetic susceptibilities for **1** under zero dc fields and (b) under 1 kOe fields 3

Figure S4 Temperature dependence of in-phase(χ') and out-of phase (χ'') ac magnetic susceptibilities under zero dc fields for **3** 3

Figure S5 (a, b) Temperature dependence and (c, d) frequency dependent of in-phase(χ') and out-of phase (χ'') ac magnetic susceptibilities under 1 kOe fields for **3** 4

Figure S6 (a) Temperature dependence of $\chi'$and χ'' ac magnetic susceptibility in 800 Hz under zero dc fields for **2** and (b) **4**. 4

S2. Supporting Tables **4**

Table S1. Dy^{III} geometry analysis of complex **1** by using SHAPE 2.1 software 4

Table S2. Dy^{III} geometry analysis of complex **3** by using SHAPE 2.1 software. 5

Table S3 Selected bond lengths [Å] and-angles [°] for complex **1** 5

Table S4 Selected bond lengths [Å] and-angles [°] for complex **2** 6

Table S5 Selected bond lengths [Å] and-angles [°] for complex **3** 7

Table S6 Selected bond lengths [Å] and-angles [°] for complex **4** 8

Table S7 Some reported Dy2 complexes exhibiting SMM behaviour. 9

References 10

S1. Supporting Figures

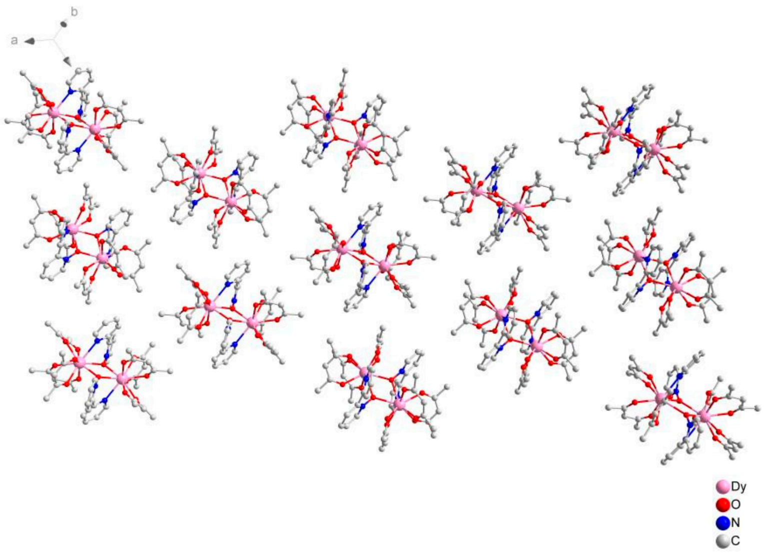

Figure S1 Crystal packing diagram of complex **1** (H and F atoms are omitted for clarity).

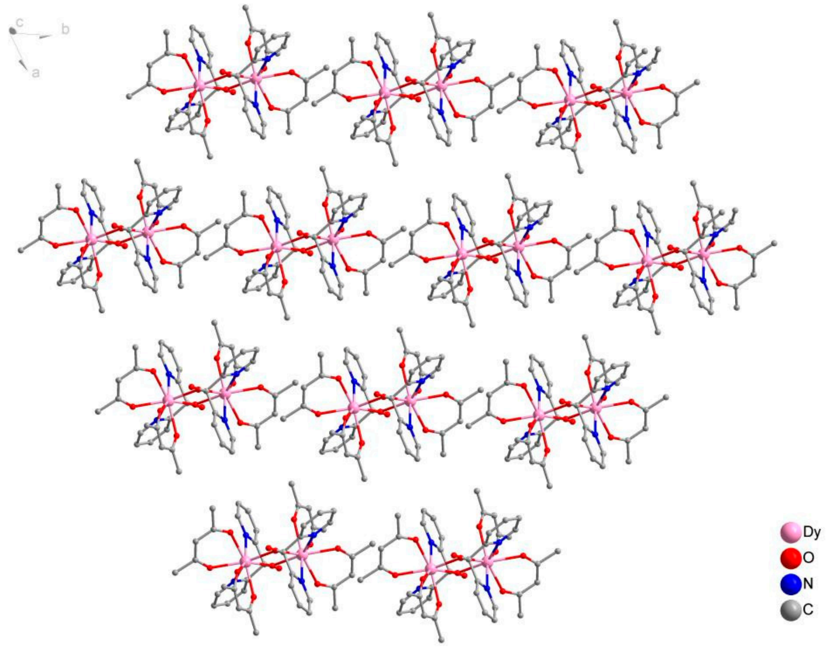

Figure S2 Crystal packing diagram of complex **3** (H and F atoms are omitted for clarity).

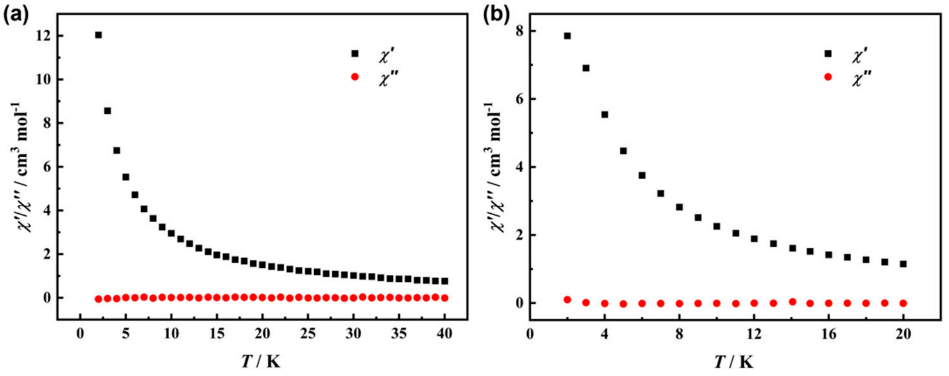

Figure S3 (a)Temperature dependence of out-of phase (χ'') ac magnetic susceptibilities for **1** under zero dc fields and (b) under 1 kOe fields

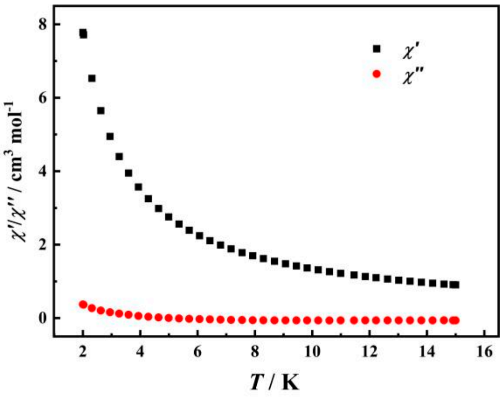

Figure S4 Temperature dependence of in-phase(χ') and out-of phase (χ'') ac magnetic susceptibilities under zero dc fields for **3**

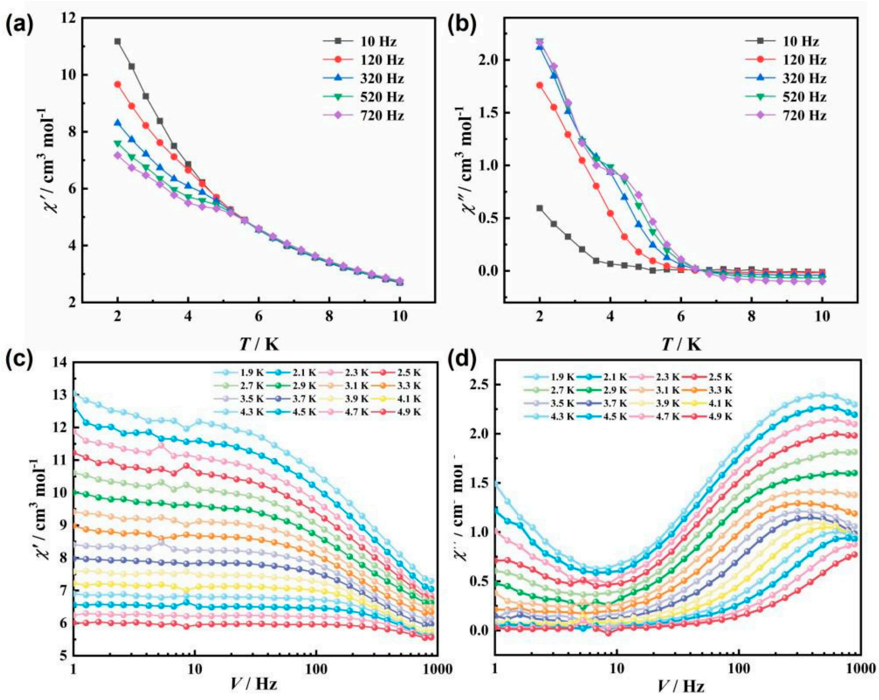

Figure S5 (a, b) Temperature dependence and (c, d) frequency dependent of in-phase(χ') and out-of phase (χ'') ac magnetic susceptibilities under 1 kOe fields for **3**

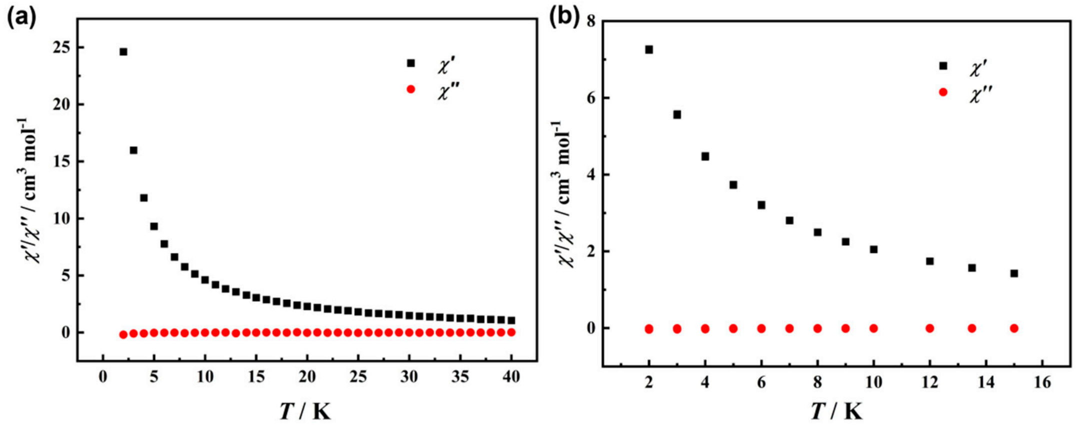

Figure S6 (a) Temperature dependence of χ' and χ'' ac magnetic susceptibility in 800 Hz under zero dc fields for **2** and (b) **4**.

S2. Supporting Tables

Table S1. Dy^{III} geometry analysis of complex **1** by using SHAPE 2.1 software

Ln^{III}	**MFF-9** (**C_s**)	**CSAPR-9** (**C_{4v}**)	**TCTPR-9** (**D_{3h}**)	**JCSAPR-9** (**C_{4v}**)	**JTCTPR-9** (**D_{3h}**)
Dy^{III}	**0.683**	1.031	1.472	2.367	2.968

MFF-9 = Muffin; CSAPR-9 = Spherical capped square antiprism; TCTPR-9 = Spherical tricapped trigonal prism; JCSAPR-9 = Capped square antiprism J10; JTCTPR-9 = Tricapped trigonal prism J51

Table S2. Dy^{III} geometry analysis of complex **3** by using SHAPE 2.1 software.

Ln^{III}	**SAPR-8** (***D*****4d**)	**TDD-8** (***D*****2d**)	**BTPR-8** (***C*****2v**)	**JBTPR-8** (***C*****2v**)	**JSD-8** (***D*****2d**)
Dy^{III}(1)	**0.814**	2.180	2.382	3.135	5.390
Dy^{III}(2)	3.186	**0.605**	2.357	3.154	2.982

TDD-8 = Triangular dodecahedron; SAPR-8 = Square antiprism; BTPR-8 = Biaugmented trigonal prism; JBTPR-8 = Biaugmented trigonal prism J50; JSD-8 = Snub diphenoid J84

Table S3 Selected bond lengths [Å] and-angles [°] for complex **1**

Bond lengths			
Dy1-O7	2.409(4)	Dy1-O6	2.368(5)
Dy1-O7[1]	2.487(4)	Dy1-O1	2.375(5)
Dy1-O3	2.487(5)	Dy1-O5	2.395(5)
Dy1-O2	2.421(5)	Dy1-N1	2.639(6)
Dy1-O4	2.332(4)	O7-Dy1[1]	2.487(4)
Bond angles			
O7-Dy1-O7[1]	58.30(16)	O1-Dy1-O7	79.69(15)
O7-Dy1-O3	119.52(15)	O1-Dy1-O3	135.78(17)
O7-Dy1-O2	129.52(17)	O1-Dy1-O2	71.07(17)
O7-Dy1-N1	64.54(16)	O1-Dy1-O5	72.82(19)
O71-Dy1-N1	84.87(16)	O1-Dy1-N1	78.63(18)
O3-Dy1-O7[1]	72.20(15)	O5-Dy1-O7	72.40(16)
O3-Dy1-N1	144.93(18)	O5-Dy1-O7[1]	90.91(17)
O2-Dy1-O7[1]	137.40(14)	O5-Dy1-O3	76.19(17)
O2-Dy1-O3	110.07(18)	O5-Dy1-O2	131.55(18)
O2-Dy1-N1	69.87(18)	O5-Dy1-N1	131.61(18)
O4-Dy1-O7	121.78(15)	Dy1-O7-Dy1[1]	121.70(16)
O4-Dy1-O7[1]	74.76(15)	N2-O7-Dy1[1]	123.1(3)
O4-Dy1-O3	68.80(17)	N2-O7-Dy1	114.6(3)
O4-Dy1-O2	67.60(17)	C19-O3-Dy1	127.5(5)
O4-Dy1-O6	96.04(17)	C14-O2-Dy1	132.4(5)
O4-Dy1-O1	137.85(17)	C17-O4-Dy1	133.0(4)
O4-Dy1-O5	144.73(17)	C24-O6-Dy1	135.1(5)
O4-Dy1-N1	79.93(16)	C12-O1-Dy1	132.5(5)
O6-Dy1-O7[1]	137.23(17)	C22-O5-Dy1	132.2(5)

O6-Dy1-O7	141.65(15)	C6-N1-Dy1	126.0(4)
O6-Dy1-O3	65.67(19)	C10-N1-Dy1	117.4(5)
O6-Dy1-O2	67.80(17)	O6-Dy1-N1	135.50(18)
O6-Dy1-O1	75.38(17)	O1-Dy1-O7[1]	137.93(15)
O6-Dy1-O5	72.65(17)		
(1): 3/2-X,1/2-Y,1-Z			

Table S4 Selected bond lengths [Å] and-angles [°] for complex **2**

Bond lengths			
Tb1-O6	2.436(4)	Tb1-O2	2.367(3)
Tb1-O4	2.349(3)	Tb1-O1	2.397(4)
Tb1-O7	2.413(3)	Tb1-O5	2.369(4)
Tb1-O7[1]	2.464(3)	Tb1-N1	2.654(4)
Tb1-O3	2.481(4)	O7-Tb1[1]	2.464(3)
Bond angles			
O6-Tb1-O7[1]	137.72(12)	O2-Tb1-O6	68.11(13)
O6-Tb1-O3	109.71(13)	O2-Tb1-O7	140.80(12)
O6-Tb1-N1	69.63(12)	O2-Tb1-O7[1]	137.56(13)
O4-Tb1-O6	67.98(12)	O2-Tb1-O3	65.74(13)
O4-Tb1-O7	121.26(11)	O2-Tb1-O1	72.62(13)
O4-Tb1-O7[1]	74.60(11)	O2-Tb1-O5	75.25(13)
O4-Tb1-O3	68.93(12)	O2-Tb1-N1	135.54(13)
O4-Tb1-O2	97.46(12)	O1-Tb1-O6	131.48(12)
O4-Tb1-O1	145.88(13)	O1-Tb1-O7[1]	90.74(12)
O4-Tb1-O5	137.79(13)	O1-Tb1-O7	71.57(12)
O4-Tb1-N1	78.87(12)	O1-Tb1-O3	77.37(13)
O7-Tb1-O6	129.38(12)	O1-Tb1-N1	131.21(13)
O7-Tb1-O7[1]	58.17(13)	O5-Tb1-O6	70.86(13)
O7[1]-Tb1-O3	72.70(11)	O5-Tb1-O7[1]	137.40(12)
O7-Tb1-O3	120.06(12)	O5-Tb1-O7	79.27(11)
O7-Tb1-N1	64.94(12)	O5-Tb1-O3	136.20(12)
O7[1]-Tb1-N1	84.74(12)	O5-Tb1-O1	72.52(13)
O3-Tb1-N1	144.41(13)	O5-Tb1-N1	78.57(13)
(1): 3/2-X,1/2-Y,1-Z			

Table S5 Selected bond lengths [Å] and-angles [°] for complex **3**

Bond lengths			
Dy1-O1	2.344(5)	Dy2-O10	2.266(5)
Dy1-O2	2.348(5)	Dy2-O10^2	2.308(5)
Dy1-O4	2.424(5)	Dy2-O6	2.341(6)
Dy1-O3	2.362(5)	Dy2-O7	2.363(5)
Dy1-O5	2.274(5)	Dy2-O8	2.354(6)
Dy1-O5^1	2.345(5)	Dy2-O9	2.360(6)
Dy1-N1^1	2.519(6)	Dy2-N4^2	2.525(7)
Dy1-N2^1	2.552(6)	Dy2-N3^2	2.548(7)
O5-Dy1^1	2.345(5)	O10-Dy2^2	2.308(5)
N1-Dy1^1	2.519(6)	N4-Dy2^2	2.525(7)
N2-Dy1^1	2.552(6)	N3-Dy2^2	2.548(7)

Bond angles			
O1-Dy1-O2	72.37(16)	O10-Dy2-O10^2	70.9(2)
O1-Dy1-O4	75.15(17)	O10^2-Dy2-O6	124.9(2)
O1-Dy1-O3	144.84(17)	O10-Dy2-O6	79.79(19)
O1-Dy1-O5^1	132.24(16)	O10^2-Dy2-O7	133.40(19)
O1-Dy1-N1^1	127.75(18)	O10-Dy2-O7	151.74(19)
O1-Dy1-N2^1	76.49(18)	O10^2-Dy2-O8	147.34(19)
O2-Dy1-O4	71.40(17)	O10-Dy2-O8	92.03(19)
O2-Dy1-O3	92.59(17)	O10^2-Dy2-O9	79.1(2)
O2-Dy1-N1^1	70.72(17)	O10-Dy2-O9	82.1(2)
O2-Dy1-N2^1	99.82(18)	O10^2-Dy2-N4^2	68.5(2)
O4-Dy1-N1^1	123.89(17)	O10-Dy2-N4^2	133.8(2)
O4-Dy1-N2^1	151.64(18)	O10^2-Dy2-N3^2	65.3(2)
O3-Dy1-O4	69.90(17)	O10-Dy2-N3^2	110.7(2)
O3-Dy1-N1^1	72.13(19)	O6-Dy2-O7	73.70(19)
O3-Dy1-N2^1	138.36(19)	O6-Dy2-O8	76.46(19)
O5-Dy1-O1	85.76(17)	O6-Dy2-O9	141.8(2)
O5-Dy1-O2	149.22(17)	O6-Dy2-N4^2	143.1(2)
O5^1-Dy1-O2	138.23(16)	O6-Dy2-N3^2	84.2(2)
O5-Dy1-O4	82.39(16)	O7-Dy2-N4^2	74.5(2)
O5^1-Dy1-O4	139.32(17)	O7-Dy2-N3^2	76.5(2)
O5-Dy1-O3	93.29(17)	O8-Dy2-O7	72.55(19)
O5^1-Dy1-O3	79.91(17)	O8-Dy2-O9	70.9(2)

O5-Dy1-O5^{1}	72.55(19)	O8-Dy2-N4^{2}	110.9(2)
O5-Dy1-N1^{1}	139.53(17)	O8-Dy2-N3^{2}	147.0(2)
O51-Dy1-N1^{1}	67.86(17)	O9-Dy2-O7	113.3(2)
O5-Dy1-N2^{1}	95.65(17)	O9-Dy2-N4^{2}	69.5(2)
O5^{1}-Dy1-N2^{1}	64.42(18)	O9-Dy2-N3^{2}	133.8(2)
N1^{1}-Dy1-N2^{1}	74.86(19)	N4^{2}-Dy2-N3^{2}	70.5(2)
C2-O1-Dy1	133.3(5)	Dy2-O10-Dy2^{2}	109.1(2)
C4-O2-Dy1	133.7(4)	C37-O10-Dy2	137.1(5)
C7-O4-Dy1	134.5(5)	C37-O10-Dy2^{2}	112.0(5)
C9-O3-Dy1	136.4(5)	C23-O6-Dy2	133.6(6)
Dy1-O5-Dy1^{1}	107.45(19)	C25-O7-Dy2	132.8(6)
C16-O5-Dy1	133.2(4)	C28-O8-Dy2	136.5(5)
C16-O5-Dy1^{1}	110.3(4)	C30-O9-Dy2	136.8(6)

(1): 1-X,-Y,2-Z; (2): -X,2-Y,1-Z

Table S6 Selected bond lengths [Å] and-angles [°] for complex **4**

Bond lengths			
N1-Tb1	2.546(9)	O5-Tb1	2.347(6)
N2-Tb1	2.547(9)	O5-Tb1^{1}	2.285(7)
N3-Tb2	2.535(11)	O6-Tb2	2.379(9)
N4-Tb2	2.576(11)	O7-Tb2	2.342(8)
O1-Tb1	2.351(7)	O8-Tb2	2.350(8)
O2-Tb1	2.367(7)	O9-Tb2	2.357(8)
O3-Tb1	2.417(7)	O10-Tb2^{2}	2.277(8)
O4-Tb1	2.375(8)	O10-Tb2	2.297(7)
Bond angles			
Tb1^{1}-O5-Tb1	107.4(3)	N3-Tb2-N4	70.6(3)
Tb2^{2}-O10-Tb2	109.2(3)	O6-Tb2-N3	69.3(3)
N1-Tb1-N2	74.4(3)	O6-Tb2-N4	134.0(3)
O1-Tb1-N1	76.1(3)	O7-Tb2-N3	110.5(3)
O1-Tb1-N2	127.6(3)	O7-Tb2-N4	146.7(3)
O1-Tb1-O2	72.5(3)	O7-Tb2-O6	70.6(3)
O1-Tb1-O3	75.2(3)	O7-Tb2-O8	72.7(3)
O1-Tb1-O4	144.7(3)	O7-Tb2-O9	77.3(3)
O2-Tb1-N1	98.9(3)	O8-Tb2-N3	74.1(3)
O2-Tb1-N2	70.6(3)	O8-Tb2-N4	76.0(3)
O2-Tb1-O3	71.3(3)	O8-Tb2-O6	112.8(3)

O2-Tb1-O4	92.5(3)	O8-Tb2-O9	74.1(3)
O3-Tb1-N1	151.2(3)	O9-Tb2-N3	142.8(3)
O3-Tb1-N2	124.0(3)	O9-Tb2-N4	83.5(3)
O4-Tb1-N1	138.9(3)	O9-Tb2-O6	142.4(3)
O4-Tb1-N2	72.6(3)	O10^2-Tb2-N3	133.5(3)
O4-Tb1-O3	69.7(3)	O10-Tb2-N3	68.5(3)
O51-Tb1-N1	96.3(3)	O10^2-Tb2-N4	110.8(3)
O5-Tb1-N1	65.2(3)	O10-Tb2-N4	65.2(3)
O5-Tb1-N2	67.9(2)	O10-Tb2-O6	79.6(3)
O5^1-Tb1-N2	139.6(3)	O10^2-Tb2-O6	82.3(3)
O5-Tb1-O1	132.2(3)	O10^2-Tb2-O7	92.5(3)
O5^1-Tb1-O1	85.5(3)	O10-Tb2-O7	147.7(3)
O5-Tb1-O2	138.1(2)	O10-Tb2-O8	132.8(3)
O5^1-Tb1-O2	149.3(2)	O10^2-Tb2-O8	152.4(3)
O5^1-Tb1-O3	82.6(2)	O10-Tb2-O9	124.2(3)
O5-Tb1-O3	139.3(2)	O10^2-Tb2-O9	80.1(3)
O5^1-Tb1-O4	93.3(3)	O10^2-Tb2-O10	70.8(3)
O5-Tb1-O4	80.0(3)	O5^1-Tb1-O5	72.6(3)
(1): 1-X,-Y,2-Z; (2): -X,2-Y,1-Z			

Table S7 Some reported Dy2 complexes exhibiting SMM behaviour.

Dy2 complex	Point Group	Magnetic coupling	The slow relaxation process			Ref.
			Field (Oe)	U_{eff} (K)	τ_0 (s)	
$[hqH_2][Ln_2(hq)_4(NO_3)_3]\cdot MeOH$	C_{2v}	F (J = 1.48 cm^{-1})	1000	59.0	1.4×10^{-6}	[1]
$[Dy_2(L^1{}_2)_2(acac)_2(C_2H_5OH)_2]$	D_{4d}	F	0	45.0	7.28×10^{-6}	[2]
$Dy_2(L^2)(Cl\text{-}salphen)_2]\cdot 0.5ClCH_2CH_2Cl$	D_{4d}	AF	2000	48.1<br>6.3	5.64×10^{-8}<br>4.80×10^{-4}	[3]
$[Dy(DBM)_2]_2(\mu\text{-}HMq)_2(n\text{-}C_6H_{14})$	C_{3v}	AF (J = -2.1 cm^{-1})	1900	18.6	2.9×10^{-6}	[4]
$[Dy(hfac)_3]_2(\mu\text{-}HMq)_2$	D_{4d}	F (J = 1.0 cm^{-1})	0	9.2	1.7×10^{-5}	[4]
$[Dy_2(hfac)_4(L^3)_2]$	D_{2d}	F	0	6.8	9.12×10^{-6}	[5]
$[Dy_2(bfac)_4(L^4)_2]\cdot C_7H_{16}$	D_{2d}	AF	0	25.7	1.64×10^{-6}	[5]
$[Dy_2(hfac)_6(BTR)]$	C_{2v}	F (J = 0.08 cm^{-1})	1000	25.3	2.60×10^{-7}	[6]

$[Dy_2(L^5)(acac)_4]$	D_{4d}	F (J = 0.72 cm^{-1})	0	66.7	1.1×10^{-8}	[7]
$[Dy_2(L^5)(tfac)_4]$	D_{4d}	F (J = 2.72 cm^{-1})	0	79.0	3.3×10^{-8}	[7]
$[Dy_2(DClQ)_6(MeOH)_2]$	C_{2v}	F (J = 0.03 cm^{-1})	0	51.4	1.1×10^{-6}	[8]
$[Dy_2(NO_3)_2(saph)_2(DMF)_4]$	D_{4d}	AF (J = -0.19 cm^{-1})	0	25.0<br>23.3	3.0×10^{-6}<br>4.4×10^{-7}	[9]
$[Dy_2(L^6R)_2(4\text{-}Me\text{-}PhO)_2(OH)_2](BPh4)_2$ (2R)	C_{2v}	F	0	138	6.3×10^{-8}	[10]
$[Dy_2(L^6S)_2(4\text{-}Me\text{-}PhO)_2(OH)_2](BPh4)_2$ (2S)	C_{2v}	F	0	160	2.1×10^{-8}	[10]

References

1. Moreno Pineda, E.; Chilton, N.F.; Marx, R.; Dörfel, M.; Sells, D.O.; Neugebauer, P.; Jiang, S.-D.; Collison, D.; van Slageren, J.; McInnes, E.J.L.; et al. Direct measurement of dysprosium(III)˙˙dysprosium(III) interactions in a single-molecule magnet. *Nature Communications* **2014**, *5*, 5243, doi:10.1038/ncomms6243.
2. Zhang, X.-M.; Duan, Y.-Y.; Gao, H.-L.; Cui, J.-Z. Solvent-induced single-molecule magnet behavior and near-infrared luminescence properties of rare earth complexes. *New Journal of Chemistry* **2020**, *44*, 19135-19143, doi:10.1039/D0NJ03837G.
3. Gao, F.; Wang, L.; Zhu, G.-Z.; Liu, Y.-H.; Yang, H.; Li, X.; Yang, K. Controllable syntheses and magnetic properties of novel homoleptic triple-decker lanthanide complexes. *Dalton Transactions* **2019**, *48*, 13360-13368, doi:10.1039/C9DT02565K.
4. Zhang, W.-Y.; Tian, Y.-M.; Li, H.-F.; Chen, P.; Sun, W.-B.; Zhang, Y.-Q.; Yan, P.-F. A series of dinuclear Dy(iii) complexes bridged by 2-methyl-8-hydroxylquinoline: replacement on the periphery coordinated β-diketonate terminal leads to different single-molecule magnetic properties. *Dalton Transactions* **2016**, *45*, 3863-3873, doi:10.1039/C5DT04449A.
5. Wang, W.-M.; Zhang, H.-X.; Wang, S.-Y.; Shen, H.-Y.; Gao, H.-L.; Cui, J.-Z.; Zhao, B. Ligand Field Affected Single-Molecule Magnet Behavior of Lanthanide(III) Dinuclear Complexes with an 8-Hydroxyquinoline Schiff Base Derivative as Bridging Ligand. *Inorganic Chemistry* **2015**, *54*, 10610-10622, doi:10.1021/acs.inorgchem.5b01404.
6. Xiao, Z.-X.; Miao, H.; Shao, D.; Wei, H.-Y.; Zhang, Y.-Q.; Wang, X.-Y. Single-molecule magnet behaviour in a dysprosium-triradical complex. *Chemical Communications* **2018**, *54*, 9726-9729, doi:10.1039/C8CC04739A.
7. Shen, F.-X.; Pramanik, K.; Brandão, P.; Zhang, Y.-Q.; Jana, N.C.; Wang, X.-Y.; Panja, A. Macrocycle supported dimetallic lanthanide complexes with slow magnetic relaxation in Dy2 analogues. *Dalton Transactions* **2020**, *49*, 14169-14179, doi:10.1039/D0DT02778B.
8. Shao, D.; Sahu, P.P.; Tang, W.-J.; Zhang, Y.-L.; Zhou, Y.; Xu, F.-X.; Wei, X.-Q.; Tian, Z.; Singh, S.K.; Wang, X.-Y. A single-ion magnet building block strategy toward Dy2 single-molecule magnets with enhanced magnetic performance. *Dalton Transactions* **2022**, *51*, 18610-18621, doi:10.1039/D2DT03046B.

9. Anastasiadis, N.C.; Kalofolias, D.A.; Philippidis, A.; Tzani, S.; Raptopoulou, C.P.; Psycharis, V.; Milios, C.J.; Escuer, A.; Perlepes, S.P. A family of dinuclear lanthanide(iii) complexes from the use of a tridentate Schiff base. *Dalton Transactions* **2015**, *44*, 10200-10209, doi:10.1039/C5DT01218J.
10. Zhao, C.; Zhu, Z.; Li, X.-L.; Tang, J. Air-stable chiral mono- and dinuclear dysprosium single-molecule magnets: steric hindrance of hexaazamacrocycles. *Inorganic Chemistry Frontiers* **2022**, *9*, 4049-4055, doi:10.1039/D2QI00754A.
